# Antimicrobial Photodynamic Approach in the Inactivation of Viruses in Wastewater: Influence of Alternative Adjuvants

**DOI:** 10.3390/antibiotics10070767

**Published:** 2021-06-24

**Authors:** Maria Bartolomeu, Cristiana Oliveira, Carla Pereira, M. Graça P. M. S. Neves, M. Amparo F. Faustino, Adelaide Almeida

**Affiliations:** 1 Department of Biology and CESAM, University of Aveiro, 3810-193 Aveiro, Portugal; maria.bartolomeu@ua.pt (M.B.); cristianapoliveira@ua.pt (C.O.); csgp@ua.pt (C.P.); 2 Department of Chemistry and LAQV-REQUIMTE, University of Aveiro, 3810-193 Aveiro, Portugal; gneves@ua.pt

**Keywords:** antimicrobial photodynamic therapy, porphyrin, wastewater, *E. coli* bacteriophage T4-like, mammalian viruses, potassium iodide, hydrogen peroxide, organic matter content

## Abstract

Pathogenic viruses are frequently present in marine and estuarine waters, due to poor wastewater (WW) treatments, which consequently affect water quality and human health. Chlorination, one of the most common methods used to ensure microbiological safety in tertiarily treated effluents, may lead to the formation of toxic chemical disinfection by-products on reaction with organic matter present in the effluents. Antimicrobial photodynamic therapy (aPDT) can be a promising disinfecting approach for the inactivation of pathogens, without the formation of known toxic by-products. Additionally, some studies have reported the potentiator effect on aPDT of some compounds, such as potassium iodide (KI) and hydrogen peroxide (H_2_O_2_). In the present study, the aPDT efficiency of a PS formulation constituted of five cationic porphyrins (Form) in the inactivation of *E. coli* T4-like bacteriophage, a model of mammalian viruses, in different aqueous matrices with different organic matter content, was evaluated. Photoinactivation studies were performed at different concentrations of Form and in the presence of the adjuvants KI and H_2_O_2_. The results showed that the efficiency of bacteriophage photoinactivation is correlated with the Form concentration, the amount of the organic matter in WW, and the adjuvant type. Form can be an effective alternative to controlling viruses in WW, particularly if combined with H_2_O_2_, allowing to significantly reduce PS concentration and treatment time. When combined with KI, the Form is less effective in inactivating T4-like bacteriophage in WW.

## 1. Introduction

The presence of pathogens in wastewater (WW) is a subject of great concern due to the impact on the quality of the receiving water where this type of effluent is discharged. The disposal of inadequately treated WW is the main source of pathogenic microorganisms in the aquatic environment [1,2,3]. Even though WW is, in general, secondarily treated before being launched into seawater and rivers, it contains high concentrations of microorganisms, but dilution makes it acceptable in terms of microbiological quality, to levels comparable to those found in natural waters, achieving the guidelines of the World Health Organization (WHO) and the United States Environmental Protection Agency (EPA) standards for microorganisms’ presence in water [4]. However, the emergence of multidrug-resistant (MDR) microorganisms and other highly pathogenic emergent microorganisms, including SARS-CoV-2, bring serious risks when WW is not properly treated, contributing to wide spread of these emerging pathogenic strains [5].

Viruses are among the most potentially hazardous pathogens found in WW [6], due to the much smaller dosages required to cause infection, when compared to other pathogenic microorganisms [6,7,8]. Additionally, viruses are inherently more resistant to adverse environmental factors and to wastewater treatment processes than bacteria [3,6,9]. Consequently, viruses have been proposed to be included alongside bacteria as indicators of the microbiological quality of different water bodies and water treatment [10,11,12]. Despite the advances in wastewater treatment plants (WWTP), a large number of human enteric viruses are discharged into the aquatic environment [9,13,14]. The most generally detected pathogenic viruses are poliovirus, enterovirus, echovirus, and coxsackievirus [6]. Genome copies of SARS-CoV-2 have also been found in untreated and treated WW [15], and a recent study has shown that infectious SARS-CoV-2 can be detected for at least seven days in WW (laboratory experiments using a virus high-titre) [16].

Conventional wastewater treatment processes are designed to reduce solids in suspension, biodegradable organic products, microorganisms, and nutrients [17]. In general, WW from urban areas is secondarily treated (rarely tertiarily) and released into seawater far from beach areas [18]. Among the tertiary treatments, chlorination was the first chemical water disinfection approach to be implemented as a standard process [19] and currently, it is the most common method of ensuring microbiological safety in tertiary effluents since it effectively inactivates bacteria and viruses [4,20]. However, its massive utilization may lead to the formation of disinfection by-products with potential health hazards, as carcinogenic chlorinated disinfection by-products are created when reacting with organic compounds present in the WW [4,19,20]. The ultraviolet (UV) light and ozonation, also used as tertiary treatments, are toxic to aquatic species, induce genetic damage to several organisms (UV light), and are highly expensive (ozonation) [18,21]. Thus, for the reduction of waterborne dissemination diseases, new and safe treatments should be developed [22,23,24].

Antimicrobial photodynamic therapy (aPDT) can be a very promising alternative to those treatments. aPDT involves the use of a photosensitizer (PS) which, in the presence of visible light and dioxygen, produces reactive oxygen species (ROS), such as free oxygen radicals and singlet oxygen (^1^O_2_). These ROS are responsible for the oxidation of several cellular/virus components (e.g., lipids and proteins from bacterial cytoplasmatic membrane and cell wall and from viral envelopes and capsids) conducting to a rapid cell/virus inactivation. The efficacy of aPDT towards viruses, as well as towards bacteria and fungi, has been extensively studied in recent years, proving to be a promising alternative to conventional antimicrobial methods. Due to its multitarget nature, the approach has a low probability of triggering the development of resistance in microorganisms [25,26,27,28], and is being considered as a promising alternative to actual methods to control water quality in different environments (e.g., aquacultures, hospital WW) [29]. The studies of aPDT against viruses began with the first reports of Schultz and Perdrau and Todd in the 1930s [30,31]. Since then, aPDT applications as an antiviral approach was mainly centered in the clinical field, in the treatment of herpes simplex virus (HSV) lesions [32,33,34,35], human immunodeficiency viruses (HIV), papillomatosis virus (HPV) [36,37], encephalomyocarditis virus (EMCV) [38], hepatitis A (HAV) [39] and hepatitis C virus (HCV) [40], as well as influenza virus [41] and enterovirus 71 [42], and even in the viral photodynamic disinfection of blood products [43,44,45]. The effect of aPDT on mammalian viruses has also been studied using bacterial viruses (bacteriophages or simply phages) as surrogates, considering that they are resistant to the water treatment and environmental factors like enteric mammalian viruses [46]. Additionally, easier procedures and more safety conditions are required to hand this type of phage when compared to active mammalian viruses [46]. The positive outcomes already achieved [20,28,39,47,48,49,50,51,52,53,54], showed that enveloped viruses are more sensitive to aPDT than the non-enveloped ones [55,56], and as most of the bacteriophages are non-enveloped viruses, they are more difficult to photoinactivate. Thus, it is expected that the establishment of an efficient aPDT protocol capable to eradicate non-enveloped bacteriophages will be most likely also efficient against both non- and enveloped mammalian viruses. Additionally, since bacteriophages are mainly DNA viruses and this type of viruses are known to be less susceptible to aPDT than their RNA counterparts [57], the protocol could be extended to any type of viruses.

An *Escherichia coli* T4-like bacteriophage, a DNA, and non-enveloped phage from the Caudovirales order, with an elongated icosahedral head and a contractile tail (*Myoviridae* family) [58] was used in the present study, as a model of enteric mammalian viruses [59,60]. Bacteriophages are frequently used as indicators of the presence of human enteric pathogens and microbial fecal pollution, which may lead to consequent public health risks. Several studies have shown a successful photoinactivation of bacteriophages [20,28,54,61], their effectiveness depending on variables such as the structural composition of the PS, including the number and position of positive charges and hydrophobicity [20], light source and total light dose [60].

**Figure 1:**
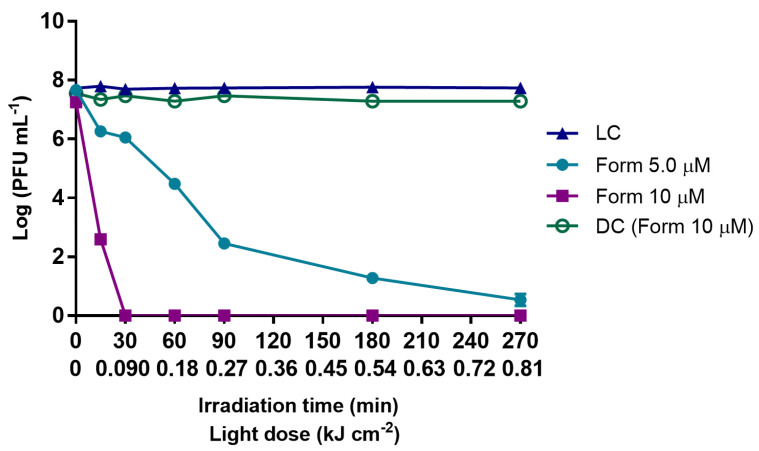
Inactivation of T4-like bacteriophage during aPDT, in PBS, with Form at 5.0 and 10 µM, for 270 min of irradiation with artificial white light (50 mW cm^−2^). The values are expressed as the mean of three independent experiments with two replicates; error bars represent the standard deviation (SD) between the experiments. In some cases, SD bars are covered behind the symbols and lines join the experimental points.

Recently, a PS formulation (Form), composed of a non-separated mixture of five cationic *meso*-tetraarylporphyrins [Mono-Py(+)-Me (19%), Di-Py(+)-Me*opp* and Di-Py(+)-Me*adj* (20%), Tri-Py(+)-Me (44%) and Tetra-Py(+)-Me (17%); Figure 1], has proven its high efficiency in the photoinactivation of microorganisms such as *Staphylococcus aureus* (a Gram-positive bacterium), *Escherichia coli* (a Gram-negative bacterium), *Pseudomonas syringae* pv. *actinidiae* (a Gram-negative bacterium), and *Candida albicans* (a fungus) [47,62,63]. This Form is being considered a relevant alternative to the highly efficient PS, the purified Tri-Py(+)-Me [64] present in its composition, due to the significant reduction in production costs and purification time consumption when compared to the included purified PSs [62,63]. However, the photodynamic efficacy of Form towards the eradication of viruses was not yet evaluated.

The use of PS combined with some inorganic salts such as sodium thiocyanate (NaSCN) [65], sodium bromide (NaBr) [66], sodium azide (NaN_3_) [67,68], and potassium iodide (KI) [69,70,71,72,73,74,75,76,77,78] was demonstrated to improve aPDT efficiency. Several in vitro and in vivo studies have shown that the addition of KI can potentiate the aPDT effect towards bacteria (such as *Acinetobacter baumannii*, *Pseudomonas aeruginosa)* and fungi (e.g., *C. albicans*) and can reduce the incidence of cell regrowth after treatment due to the production of free iodine/triiodide (I_2_/I_3_^−^), iodine radicals (I_2_^•−^) and hydrogen peroxide (H_2_O_2_), longer-lived reactive species than ^1^O_2_ that may remain active even after the aPDT treatment [64,69,70,71,72,73,74,75,76,77,78,79,80]. In 2018, Vieira et al. [64] reported that the combination of Form with KI was highly efficient in the photoinactivation of *E. coli*, when compared to the photoinactivation of this bacterium in the presence of Form alone.

According to some authors, hydrogen peroxide (H_2_O_2_) can also be effectively combined with PS as an enhancer of aPDT effectiveness [81,82]. The radical species produced from the PS irradiation would react with H_2_O_2_ producing hydroxyl radicals [83], thereby increasing the number of free radical species available in solution to damage viral structures as proteins, lipids, and nucleic acids [84,85,86].

All of this prompts us to evaluate the photodynamic effect of Form alone and in the presence of co-adjuvants such as KI and H_2_O_2_ in the inactivation of bacteriophages in WW. The experiments were carried out in filtered and non-filtered WW matrixes loaded with *E. coli* T4-like bacteriophage. The results obtained in these matrixes with different organic matter content were compared with the ones obtained in phosphate-buffered saline (PBS) used as a standard aqueous matrix.

## 2. Results

### 2.1. aPDT Assays in PBS

The results summarized in Figure 1 show that when Form was used in PBS at 5.0 μM, a controlled microcosm condition, the content in bacteriophage decreased more than 7 log PFU mL^−1^ after 270 min of treatment (0.81 kJ cm^−2^ of total light dose) when compared to the sample before aPDT (*p* value < 0.0001), the most abrupt decrease occurring in the first 90 min of irradiation (0.27 kJ cm^−2^ of total light dose) with inactivation of ca. 5 log PFU mL^−1^ (*p* value < 0.0001). When Form concentration was doubled (10 μM), the inactivation efficiency was greatly increased and inactivation of more than 7 log PFU mL^−1^ (detection limit of the method) was reached just after 30 min (0.09 kJ cm^−2^ of total light dose) of irradiation (Figure 1). The Form concentration had a significant effect on the bacteriophage inactivation rate; the comparison of the reduction obtained after 30 min of treatment (0.09 kJ cm^−2^) with Form at 5.0 μM (1.4 log PFU mL^−1^), with the one with Form at 10 µM (>7 log PFU mL^−1^) showed a sharp difference of 6 log PFU mL^−1^ (*p* value < 0.0001). Both light (LC) and dark [DC (Form)] controls remained constant along the experiment period, meaning that neither white light radiation alone nor Form at the highest tested concentration (10 μM) in the dark have a toxic effect on the bacteriophage particles.

To evaluate the possible effects on the content in bacteriophage by reactive species formed when KI reacts with ^1^O_2,_ namely free iodine/triiodide (I_2_/I_3_^−^), iodine radicals (I_2_^•−^) and hydrogen peroxide (H_2_O_2_) with longer lifetimes than ROS in PBS, bacteriophage suspension in PBS was treated with Form at 5.0 (see Supplementary Materials, Figure S1A) and 10 µM (See Supplementary Materials, Figure S1B), irradiated for 15 min (sublethal aPDT treatment) and then kept in the dark. In these assays, when KI was added to the bacteriophage suspension, a slower bacteriophage inactivation was observed after bacteriophage quantification immediately after the aPDT treatment (*ca*. 0.74 log PFU mL^−1^ vs. ca. 3.0 log PFU mL^−1^ of bacteriophage inactivation for Form at 5.0 µM with KI at 100 mM and without KI, respectively, and ca. 0.62 log PFU mL^−1^ vs. ca. 4.8 log PFU mL^−1^ of bacteriophage inactivation with Form at 10 µM with KI at 100 mM and without KI, respectively). Moreover, when the content of bacteriophage in a further period after this aPDT treatment (15 min of irradiation) was quantified, the results did not show any significant inactivation effect along the further dark incubation period (*p* value > 0.05) for the Form alone (at 5.0 and 10 µM) nor Form combined with KI at 100 mM. In fact, the sample with Form plus KI does not show any decrease in the bacteriophage content along the same dark incubation period, suggesting that the combination of Form plus KI (even potentially resulting in the formation of highly reactive species with longer half-time) had no prolonged inactivation effect during the dark incubation nor does it act on the viral particles.

### 2.2. aPDT Assays in Filtered Wastewater

**Figure 2:**
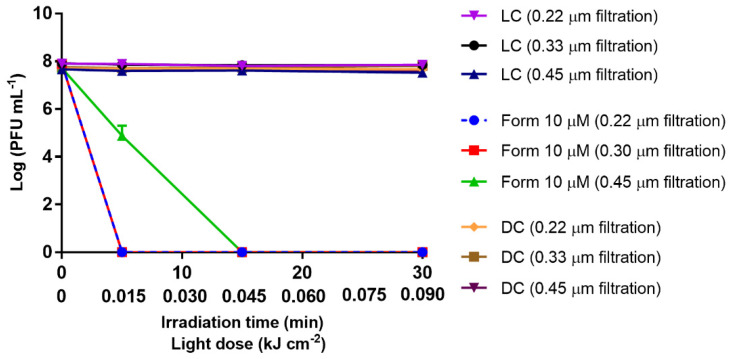
Inactivation of T4-like bacteriophage during aPDT, in filtered WW (0.22, 0.30, and 0.45 µm pore size filtration) with Form at 10 µM, for 30 min of irradiation with white light (50 mW cm^−2^). The values are expressed as the mean of three independent experiments; error bars represent the standard deviation (SD) between the experiments. In some cases, SD bars are covered behind the symbols and lines just join the experimental points.

To evaluate the influence of the organic matter present in WW in the aPDT efficiency, three different wastewater samples were filtered using three different pore size membranes (0.22, 0.30, and 0.45 µm). Thus, in the aPDT assays performed in WW filtered through pore-size membranes of 0.20 and 0.30 μm and treated with Form at 10 μM, it was observed that the efficiency of the bacteriophage inactivation was significantly improved (*p* value < 0.0001) (Figure 2), when compared with the previous results in PBS at the same concentration; in both matrixes, the reduction of phage viability attained the limit of detection of the method after 5 min of treatment (0.015 kJ cm^−2^ of total light dose) vs. the 30 min (0.09 kJ cm^−2^ total light dose) in PBS. Additionally, inactivation assays were also conducted in 0.45 μm filtered WW to minimize the dissolved organic matter suppression (maintaining most of the organic content), while allowing the removal of most of the particulate organic matter, such as the microorganisms naturally present in WW. The bacteriophage reduction to the detection limit of the method in WW filtered by 0.45 µm required 15 min (0.045 kJ cm^−2^) of aPDT treatment with Form at 10 μM (*p* value < 0.0001), showing that the amount of remained dissolved organic matter would affect aPDT efficiency (Figure 2). However, when comparing the inactivation results, in WW (0.22, 0.30, and 0.45 µm) with that in PBS, at the same Form concentration (10 μM), in all WW samples, the inactivation occurred sooner (5–15 min) than in the PBS-controlled microcosm (30 min), showing that in filtered WW, regardless of the pore size membrane used, the bacteriophage inactivation was more efficient than in PBS.

**Figure 3:**
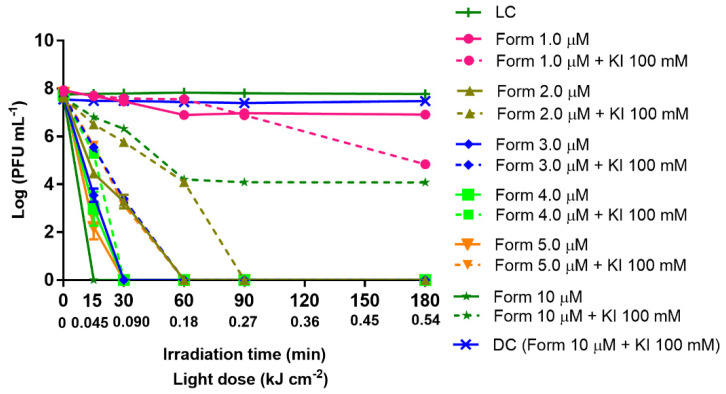
Inactivation of T4-like bacteriophage during aPDT, in filtered WW (0.45 µm pore size filtration) with Form at different concentrations (1.0, 2.0, 3.0, 4.0, 5.0, and 10 µM) alone and in combination with KI (at 100 mM), for 180 min of irradiation with white light (50 mW cm^−2^). The values are expressed as the mean of three independent experiments; error bars represent the standard deviation (SD) between the experiments; to note, in some cases, SD bars are covered behind the symbols and lines just join the experimental points.

It is known that KI can potentiate the PS inactivation effect on bacteria. Thus, the aPDT treatment with Form at concentrations ranging from 1 to 10 μM (1.0, 2.0, 3.0, 4.0, 5.0, and 10 μM) was performed in the absence and presence of KI (100 mM) and summarized in Figure 3, with 0.45 μm filtered WW. The 0.45 μm filtered WW samples were selected when they required a longer aPDT treatment to reach the detection limit of the method (15 min). In all the experiments, both light [LC and LC (KI)] and dark controls [DC and DC (Form + KI)] remained constant along the experiment period, meaning that Form plus KI in the dark or KI in the presence of light have no toxic effect on the T4-like bacteriophage particles and that the white light radiation alone has no effect on the viral particles’ viability.

The T4-like bacteriophage inactivation with Form alone (without KI addition) was demonstrated to be concentration-dependent, the detection limit of the method being reached after 15 min of irradiation when Form was used at 10 μM, 30 min of irradiation in the presence of Form ranging from 3.0 to 5.0 μM, and 60 min of irradiation with Form at 2.0 μM. At a concentration of 1.0 of Form, a reduction of only 2.9 log PFU mL^−1^ was observed after 180 min of aPDT treatment (Figure 3). Moreover, when Form was used in combination with KI at 100 mM, in general, the results showed that the use of KI does not potentiate the aPDT efficiency to photoinactivate T4-like bacteriophage in filtered WW. Moreover, the retarding effect of KI on aPDT inactivation efficiency was either significant (*p* value < 0.0001) or did not promote any beneficial effect whatsoever. Thus, T4-like bacteriophage inactivation to the detection limit of the method was observed only at 3.0 and 5.0 μM after 60 min of irradiation, while 90 min of irradiation was needed when Form was assessed at 2.0 μM in combination with KI. For Form at 1.0 μM with the addition of KI, a reduction of 0.85 log PFU mL^−1^ was observed after 180 min, but the detection limit of the method was not reached.

In addition, to evaluate if the action of the long-lived reactive species formed when KI reacts with ^1^O_2_ (e.g., I_2_/I_3_^−^, I_2_^•−^ and H_2_O_2_) was extended beyond the aPDT treatment in WW, the assays performed in 0.45 µm filtered WW in the presence of Form at 5.0 µM and KI at 100 mM were irradiated for 15 min and then kept in the dark for a maximum of 90 min and then outcomes were analyzed (See Supplementary Materials, Figure S2); for comparison, a similar analysis was performed in the absence of KI. The T4-like bacteriophage content in WW during a period ranging from 0–90 min after the 15 min of irradiation (See Supplementary Materials, Figure S2A) showed no significant effect along the dark incubation period (*p* value > 0.05) for the Form alone and the Form combined with KI. The extension of the period of irradiation to 30 min revealed a similar bacteriophage content profile to the 15 min of irradiation (See Supplementary Materials, Figure S2B).

### 2.3. aPDT Assays in Non-Filtered Wastewater

**Figure 4:**
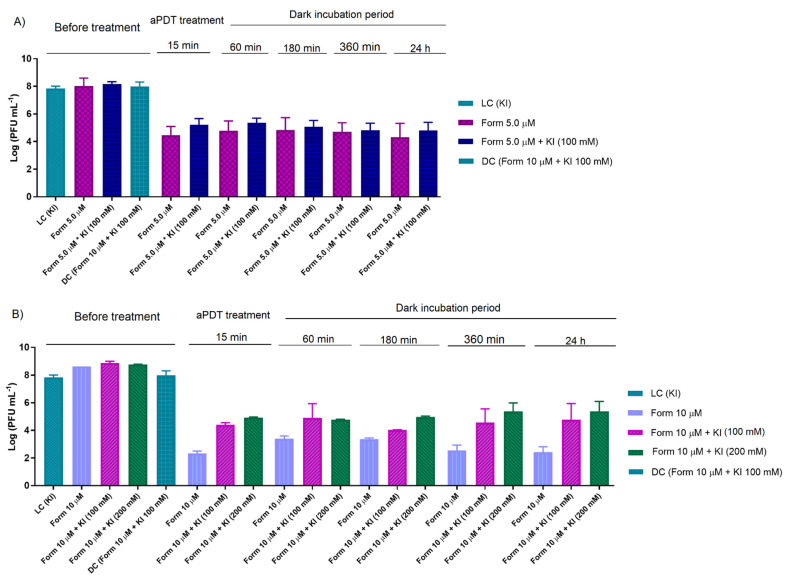
Effect of residual iodine species in dark incubation after aPDT assay in T4-like bacteriophage in non-filtered WW with Form at 5.0 and KI at 100 mM (**A**), and 10 µM and KI at 100 and 200 mM (**B**), during 15 min of irradiation with white light (50 mW cm^−2^). The values are expressed as the mean of three independent experiments; error bars represent the standard deviation (SD) between the experiments. In some cases, SD bars are covered behind the bars.

**Figure 5:**
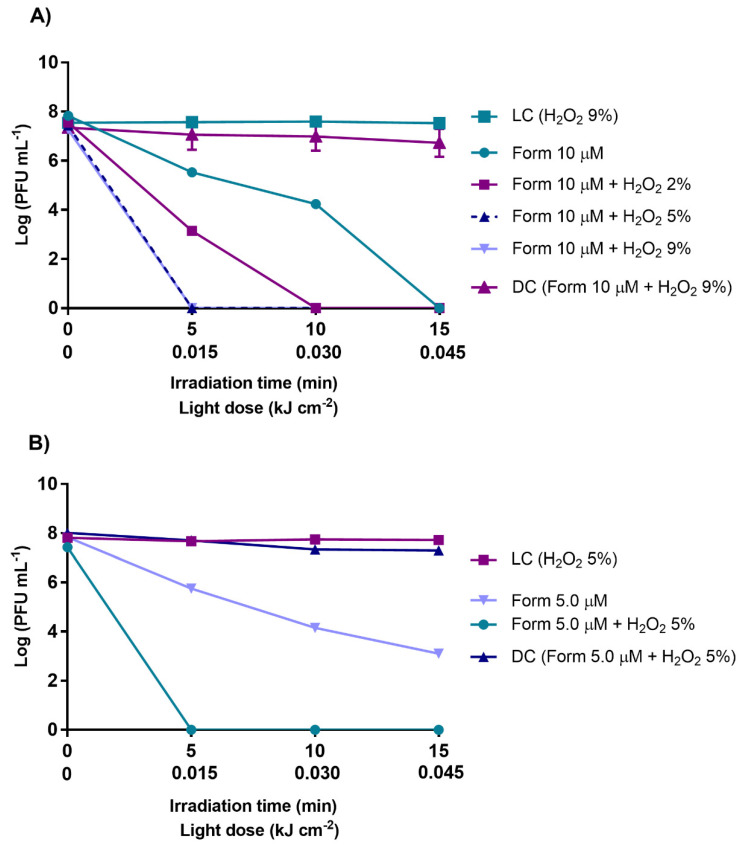
Inactivation of T4-like bacteriophage during aPDT, in non-filtered WW with Form at 10 µM alone and in combination with H_2_O_2_ (2, 5, and 9%) (**A**), and Form at 5.0 µM alone and in combination with H_2_O_2_ (at 5%) (**B**), during 15 min of irradiation with white light (50 mW cm^−2^). The values are expressed as the mean of three independent experiments; error bars represent the standard deviation (SD) between the experiments. In some cases, SD bars are covered behind the symbols and the lines just join the experimental points.

In the aPDT assays performed in non-filtered WW, the irradiations were performed in the presence of Form at 5.0 and 10 μM in the absence and the presence of KI (Figure 4) and also in the presence of H_2_O_2_ (Figure 5). It is worth noting that in these experiments, the secondarily treated WW was not filtered before the assays and some short differences in the inactivation times are easily explained by differences in the chemical composition of the WW samples, since they were collected on different days (Figure 4 and Figure 5).

Besides, the protocol experiments were extended beyond the irradiation period of the aPDT treatment as previously (0.45 μm filtered WW) to evaluate the action of the long-lived reactive species formed by the reaction of KI with ^1^O_2_. Thus, Form at 5.0 (Figure 4A) and 10 μM (Figure 4B) in non-filtered WW was irradiated for 15 min and then kept in the dark for a maximum period of 24 h. However, the KI addition showed no significant effect along the dark incubation period for the Form combined with KI at 100 (for Form at 5.0 and 10 μM) and 200 mM (only for Form at 10 μM), as seen in the same type of experiments in PBS.

These results confirm that when WW was used as an aqueous matrix, there was a lack of potentiation from longer-lived reactive species formed from KI (e.g., iodine species) during the dark incubation period after the PDT treatment.

Considering the importance of improving the aPDT wastewater protocol to inactivate the T4-like bacteriophage, the studies in non-filtered WW were extended to combinations of Form plus hydrogen peroxide (H_2_O_2_) since this agent is also recognized to be able to potentiate the photodynamic action of some PS [82,83].

Under this context, the effects of aPDT with Form at 10 μM was evaluated with the potentiator H_2_O_2_ ranging from 2.0 to 9.0% (Figure 5A). For Form at 5.0 μM, the H_2_O_2_ effect was evaluated at a concentration of 5.0% (Figure 5B). For aPDT experiments with Form at 10 μM, improved results were observed with and without the addition of H_2_O_2_ (2.0, 5.0, and 9.0%, of H_2_O_2_) (*p* value < 0.0001). The detection limit of the phage (more than 7 log PFU mL^−1^) was reached just after 5 min of treatment (15 J cm^−2^ light dose) with Form and H_2_O_2_ at 5 and 9%, and after 10 min with Form and H_2_O_2_ at 2.0%, compared to Form alone (at 10 μM), where the detection limit was reached only after 15 min of treatment. Similar results were observed with Form at 5.0 μM in combination with H_2_O_2_ at 5.0% in non-filtered WW an increase in phage inactivation was observed, significantly enhancing the inactivation efficiency of Form. Bacteriophage reductions of ca. 4 log PFU mL^−1^ was seen when the Form was used alone at 5.0 μM and 15 min of irradiation, and after the addition H_2_O_2_ at 5.0%, the detection limit of the method (>7 log PFU mL^−1^) was reached just after 5 min of treatment (0.015 kJ cm^−2^) (*p* value < 0.0001). Similarly, in the light [LC (H_2_O_2_)] and dark controls [DC (Form + H_2_O_2_)], no decrease in phage concentration was detected. These results indicate that the viability of this bacteriophage was not affected by irradiation, nor by the presence of the H_2_O_2_ or by any of the tested combinations of Form plus H_2_O_2_ in the dark.

The results of 15 min of irradiation with Form at concentrations of 5.0 and 10 μM, for the used aqueous matrices (non-filtered WW, filtered WW by 0.45 μm pore size membrane and PBS), showed different phage inactivation efficiencies. It is shown that there was a greater inactivation when the assays were done in filtered WW compared to both aqueous matrices PBS and non-filtered WW, for the two Form concentrations. The particulate organic matter seems to have a slowing effect on phage photoinactivation (filtered WW compared to non-filtered WW). However, dissolved organic matter, and other compounds that may be dissolved in the WW, appears to have a beneficial effect on phage photoinactivation (in comparison, inactivation was much higher/faster in filtered WW than in both PBS and non-filtered WW matrices).

## 3. Discussion

The photoinactivation of non-enveloped viruses occurs mainly due to the generation of ROS that interacts with the external proteins of the capsid, through degradation, as well as cleavage and cross-linking modifications, among others, leading to damages in fundamental structural and functional molecules [84]. Particularly, to T4-like phage, degradation of several proteins, including a long-tail fiber protein, was observed, by Costa et al., 2014. This protein is involved in the interaction with specific receptors on the cell host surface, and so, in the host infection process [84]. Damages in the specific proteins responsible for the host recognition as infection were also observed for other phages [87,88]. Still, the efficiency of the process has shown to be highly dependent on some factors as the number and position of charges of the porphyrins and the composition of the substituents in the *meso*-positions of the porphyrin macrocycle [54]. However, the efficiency of aPDT in more complex matrices can be also affected by the presence of particulate and dissolved organic matter, and chemicals, among other factors [24,89,90,91].

Although all the five cationic porphyrins included in the used formulation (Form) are able to be generate with high efficiency ^1^O_2_ [92], this ROS is known to have a short life span due to its unstable electronic configuration, which consequently leads to a small range diffusion ability. However, the range diffusion of ^1^O_2_ or other ROS is also highly dependent on the type of environment. Since there is a large amount of organic matter in WW, it may allow the appearance of different microenvironments with different diffusion rates [86]. Moreover, the presence in the dissolved matter of natural PSs capable of exerting their photodynamic action either through a direct reaction with their electronically excited triplet states or with the additional generation of ROS may lead to an improvement in the aPDT efficiency of the microcosm [89,93]; on the other hand, the presence of particulate organic matter can easily quench or scavenge ROS or even the PS, decreasing the PS availability to generate ROS, or the ROS availability to interact with the viral particles. These events may affect bacteriophage inactivation and, consequently, be responsible for the observed differences in the bacteriophage T4-like photoinactivation rates between assays performed in PBS and in filtered and non-filtered secondarily treated WW.

Some previous studies [91,94], using Tri-Py(+)-Me (one of the PSs included in the Form composition, used in the present work) as PS against several bacteria, had shown that aPDT efficiency was higher when performed in PBS, comparing its effectiveness in aquaculture water. However, in this study when aPDT was performed in WW (filtered and non-filtered), the bacteriophage inactivation was more effective than when aPDT was performed in PBS (Figure 1, Figure 2, Figure 3, Figure 4 and Figure 5). The inactivation to the detection limit was reached after 15 min (0.045 kJ cm^−2^ total light dose) in WW (in 0.45 µm filtered WW and in non-filtered WW), and only after 30 min (0.090 kJ cm^−2^) in PBS, using the same PS concentration (10 µM). In fact, in 2014, Almeida and co-workers [90] performed aPDT against bacteria in hospital WW, resulting in a higher inactivation efficiency (during the initial period of treatment of 30 min, 0.0072 kJ cm^−2^ total light dose) in the hospital WW when compared with PBS (a difference of ca. 2 log colony-forming unit (CFU) per mL). In addition, as previously suggested [24,90], suspended and dissolved organic matter present in the aqueous matrix may act as an aPDT efficiency enhancer, positively influencing the PS activity, possibly by the presence of other compounds that may be found in WW, as pharmaceutical compounds, and detergents. Likewise, the same authors [24,90] justified this behavior due to the presence of organic matter that can act as a factor that influences the efficiency of the PS in aPDT treatment. However, in those studies [24,90] and the current one, since no changes in bacteriophage viability were observed neither in the light and dark controls, it can be inferred that the dissolved compounds do not directly affect the viability of the bacteriophage, but it leaves the possibility that the interaction of the formed ROS with the organic matter present in the environment can lead to the formation of compounds with antimicrobial activity. It has been pointed out that the effect of photodynamic action involves the organic matter content alongside the three main actors of the “photodynamic trinity” (visible light, dissolved dioxygen, and an appropriate PS) [95]. Once organic matter is present in the reaction medium at high concentrations, the electronically excited PS can easily interact with it (Table 1), generating short-lived intermediates who consequently will interact with dissolved dioxygen, or even the excited PS can directly interact with O_2_. By any of the reaction routes, high ROS are formed, and unsaturated organic compounds can be oxidized to peroxides [96], which by further photochemical decomposition can originate free radical oxidation reactions, affecting saturated compounds that may be present in the WW [95]. As a consequence, in such a heterogeneous system, more ROS photoproduction can occur, resulting in a higher inactivation rate. As for these PSs, the most predominant pathway in their photodynamic action is via energy transfer [57,92,97] and taking into account the experimental conditions in which the assays were performed (light irradiance, time exposure of the samples to irradiation, incubation temperature), a close interaction between ^3^PS^*^ and organic matter, resulting in electron transfer, must not have caused a significant depletion of PS via chemical reaction and (photo)degradation of PS [27,98].

In this study, when the content of particulate organic matter in the aqueous matrix was reduced, the aPDT efficiency had a slight increase. About 8 log PFU mL^−1^ was inactivated after 5 min of treatment (0.015 kJ cm^−2^) in the samples using 0.22 and 0.33 µm filtered WW when compared with the 0.45 µm filtered WW and the non-filtered WW samples, for which the 8 log PFU mL^−1^ reduction was reached only after 15 min of treatment (0.045 kJ cm^−2^) (Figure 1 and Figure 2). These results were also found in similar studies previously performed [24], where the presence of organic matter may have interfered with the efficiency of photoinactivation, once most of the particulate organic matter is removed (maintaining the dissolved organic matter in the reaction medium) by filtration, reducing the turbidity of the water samples and, consequently, increasing light penetration, allowing the light to penetrate deep into the water column, resulting in a higher amount of PS molecules to be activated, and ROS to be formed.

When aPDT was performed in filtered WW (by 0.45 µm pore size membrane) and various PS concentrations were tested with and without the presence of KI, the minimum Form concentration that allowed significant inactivation to 30 min of treatment (within the several concentrations tested, ranging from 1.0 to 10 µM) was 3.0 µM. With this concentration, the detection limit of the method (reduction of 8 log PFU mL^−1^) was reached after 30 min of irradiation (0.090 kJ cm^−2^ light dose), against 15 min of treatment when Form at 10 µM was used (Figure 3). When aPDT was performed in the presence of the adjuvant KI (at 100 mM), contrary to our expectation, no potentiation effect occurred. The inactivation in the samples with Form plus KI was reached in general later when compared with the samples at the same Form concentration but without KI. In fact, some previous studies demonstrated that KI enhances aPDT efficiency against a variety of microorganisms, as Gram-negative and Gram-positive bacteria, and fungi [64,74,99]. However, in a more recent study, both effects were demonstrated—the potentiation and non-potentiation effect of KI in microorganism’s inactivation. The potentiator effect of the KI was demonstrated against both Gram-positive (*S. aureus*) and Gram-negative bacteria (*E. coli*) and a fungus (*C. albicans*), with several Form concentrations (between 0.5 and 5.0 µM) (Vieira et al., 2019). However, the effect of Form with the addition of KI did not increase the aPDT efficiency of the T4-like bacteriophage, or in PBS as the used aqueous matrices [47].

With the purpose of mimicking a scenario as close as the reality of an effluent reaching the tertiary treatment in a WWTP, non-filtered WW was used. In this scenario, since all the organic matter, chemical compounds, and various microorganisms are present, the Form concentration was used at 5.0 and 10 µM (Figure 5). After 15 min of light exposure, a bacteriophage content decrease of ca. 8 log PFU mL^−1^ was reached, attaining the detection limit of the method. These results were very similar to those obtained in 0.45 μm filtered WW and significantly better than those obtained in PBS (30 min) (*p* > 0.001), which reveals that the organic matter present in both matrices must be relevant for the inactivation of the bacteriophage. Our results are in accordance with previous experiments performed in hospital WW [90], in which aPDT efficiency was higher in the hospital WW matrix compared to PBS. As suggested for the hospital WW study [90], in our case, the presence of organic matter and chemical compounds seems to increase the effectiveness of aPDT.

With the aim of searching for an alternative to improve the efficiency of aPDT in non-filtered WW, experiments were conducted with a combination of Form and H_2_O_2_. Hydrogen peroxide is already used worldwide in the clinical field due to its sharp oxidizing properties [83]. This oxidant is commercialized at concentrations of 30 and 9.0%. Thus, tests were performed with concentrations equal to and lower than 9% to be in accordance with what is recommended for human use to prevent any risk of toxicity [100]. For the tested concentrations of 2.0, 5.0, and 9.0% of H_2_O_2_ combined with Form at 10 µM, 5.0% of H_2_O_2_ was the lowest concentration with the best performance for the combination of Form + H_2_O_2_ (Figure 5). A reduction to the detection limit of the method (reduction of about 7.5 log PFU mL^−1^) was reached at 5 min (0.015 kJ cm^−2^ light dose) of treatment. Thus, in non-filtered WW, it was possible to reduce the treatment time up to 1/3 relatively to the time required of aPDT treatment in the presence of Form alone. Additionally, when Form was used at half the concentration, 5.0 µM (alone achieved reductions of ca. 4 log PFU mL^−1^ after 15 min of irradiation), with the addition of the non-toxic H_2_O_2_ at 5.0%, the bacteriophage inactivation rate (limit of detection reached after 5 min of irradiation) was the same to that of Form at 10 µM in the presence of H_2_O_2_ at 5.0%, showing that the addition of H_2_O_2_ to the system brings a huge improvement to aPDT effectiveness in the inactivation of the bacteriophage, improving the action of the PS Form. Parallelly, in light and dark controls, containing H_2_O_2_ at the highest concentration (9.0%), no effects on the bacteriophage viability were detected, showing that the H_2_O_2_ alone, at the allowed concentration, does not promote bacteriophage inactivation. As previously mentioned by Awad et al. (2013), H_2_O_2_ alters the microbial external structures’ permeability. This effect might allow PS accumulation in the viral particles and/or increase in the ROS penetration, increasing viral inactivation. The presence of H_2_O_2_ and its photodecomposition might also increase the dioxygen availability, consequently increasing the ROS formation [83,101], and thus the viral inactivation.

## 4. Materials and Methods

The effectiveness of aPDT against the phage was evaluated using three different types of microcosms: (i) phosphate-buffered saline (PBS); (ii) filtered WW; (iii) non-filtered WW. The first microcosm (PBS) was used as the standard condition. Buffered solutions, such as PBS, are useful to evaluate the behavior and efficacy of the PSs in a medium without the interference of organic matter to select the best aPDT conditions. However, as the composition of the test matrix is an influencing factor for aPDT efficiency, to pave a realistic application, it is required to test the aPDT protocol in a relevant setting, such as is the case of this study, in filtered and non-filtered WW.

In filtered WW, the assays were carried with different pore size membranes (0.22, 0.30, and 0.45 µm) to evaluate the effect of the dissolved organic matter content in the efficiency of aPDT protocol. In addition, a wide range of Form concentrations (from 1.0 to 10 µM) with and without the addition of KI (100 mM) were tested in WW filtered through a 0.45 µm pore size membrane to maintain most of the dissolved organic content (minimizing the dissolved organic matter suppression but removing the particulate organic matter) and at the same time to allow the removal of most of the microorganisms naturally present in WW. Then, the possible extended effect of longer-lived reactive species such as I_2_/I_3_^−^, I_2_^•−^ was evaluated during dark incubation after the aPDT protocol.

Lastly, the potentiator effect of H_2_O_2_ added to Form was tested in non-filtered WW.

### 4.1. Wastewater Samples

Secondarily treated WW composite samples were collected at a wastewater treatment plant (WWTP) located at the littoral center of Portugal. This facility serves a wide geographic area, which encompasses several industrial as well as urban areas served by a sanitary network. Composite samples were representative of a period of 24 h and were collected on different days. Samples were collected in the early morning, protected from light, and refrigerated at 4 °C, encompassing nine months in total from the first to the last collection (from October 2018 until October 2020). Depending on the purpose of the assays, some of the collected samples were filtered using sterile 0.22, 0.30, and 0.45 μm pore-size membranes (Millipore, Bedford, MA, USA), to eliminate residual bacteria and some particulate organic material.

### 4.2. Bacterial Strain and Growth Conditions

*E. coli* (ATCC 13706) was used in this study as the *E. coli* T4-like bacteriophage host. Fresh bacterial culture was maintained in Tryptic Soy Agar medium (TSA, Liofilchem, Italy) at 4 °C. Before each assay, one isolated colony was aseptically transferred to 30 mL of Tryptic Soy Broth medium (TSB, Liofilchem, Italy) and grown overnight at 37 °C under stirring (120 rpm). An aliquot (300 μL) of the previously mentioned culture was transferred into 30 mL of fresh TSB under the same prior growth conditions to reach the stationary phase of approximately 10^8^–10^9^ colony-forming units per mL (CFU mL^−1^).

### 4.3. Bacteriophage Preparation

A T4-like bacteriophage (phage phT4A), previously isolated from a sewage network of Aveiro, using *E. coli* as the host [20], was used. The phage suspensions were prepared from the phage stock previously prepared in SM buffer [0.1 M NaCl (Merck KGaA, Darmstadt, German), 8 mM MgSO_4_ (Merck KGaA), 20 mM Tris-HCl (Merck KGaA), 2% (*w*/*v*) gelatin, pH 7.5]. Three hundred microliters of the phT4A phage stock were added to 30 mL of *E. coli* culture in the exponential growth phase. The suspension was grown overnight and incubated at 25 °C at 50 rpm. The lysates were incubated with chloroform (final volume of 1%) for 1 h at 120 rpm. After incubation, the lysate was centrifuged at 10,000× *g* for 10 min at 4 °C to remove intact bacteria or bacterial debris. Phage suspension was stored at 4 °C and the titer was determined by the double-layer agar method [102]. Successive dilutions of the phage suspension were performed in phosphate-buffered saline solution [PBS, 137 mM NaCl (Merck KGaA), 2.7 mM KCl (Merck KGaA), 8.1 mM Na_2_HPO_4_.2H_2_O, 1.76 mM KH_2_PO_4_ (Merck KGaA), pH 7.4], and 500 µL of each dilution, together with 200 µL of fresh bacterial culture, and were mixed with 5 mL of TSA-soft [TSB 0.6% top agar layer (30 g L^−1^ TSB (Liofilchem), 6 g L^−1^ agar (Liofilchem), 0.05 g L^−1^ CaCl_2_ (Merck KGaA), 0.12 g L^−1^ MgSO_4_ (Merck KGaA), pH 7.4)] and placed over a TSA plate. The plates were incubated at 37 °C for 12 h. After incubation, the number of plaques was counted, and the results expressed as plaque-forming units per milliliter (PFU mL^−1^).

### 4.4. Antimicrobial Photodynamic Therapy (aPDT) Procedure

#### 4.4.1. Photosensitizer

**Figure 6:**
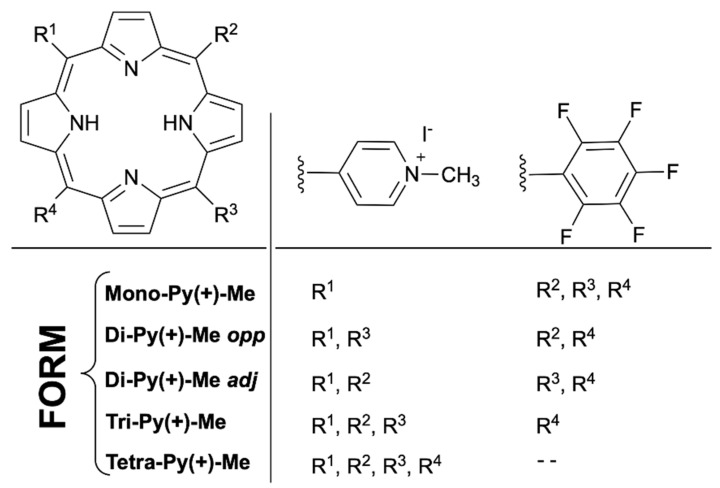
The chemical structure and respective acronyms of the porphyrin derivatives composing the non-separated mixture of *meso*-tetraarylporphyrins, named as Form in the present work.

A stock solution of Form was prepared at 500 µM in dimethyl sulfoxide (DMSO) and kept at room temperature and protected from light. Form is a non-separated mixture of five *meso*-tetraarylporphyrins positively charged (Figure 6), composed by 5-(1-methylpyridinium-4-yl)-10,15,20-tris(pentafluorophenyl)porphyrin mono-iodide [Mono-Py(+)-Me], 5,15-bis(1-methylpyridinium-4-yl)-10,20-bis(pentafluorophenyl)porphyrin di-iodide [Di-Py(+)-Me*opp*] 5,10-bis(1-methylpyridinium-4-yl)-15,20-bis(pentafluorophenyl)porphyrin di-iodide [Di-Py(+)-Me*adj*], 5,10,15-tris(1-methylpyridinium-4-yl)-20-(pentafluorophenyl)porphyrin tri-iodide [Tri-Py(+)-Me] and 5,10,15,20-tetrakis(1-methylpyridinium-4-yl)porphyrin tetra-iodide [Tetra-Py(+)-Me] and was synthetized according to the literature [62,92]. Before each assay, the stock of Form solution was sonicated for 30 min at room temperature (ultrasonic bath, Nahita 0.6 L, 40 kHz) to ensure the homogeneity of the solution, and then the appropriate volume was added to the assay’s suspension.

#### 4.4.2. Potassium Iodide and Hydrogen Peroxide Solutions Preparation

The solutions of potassium iodide (KI) (Merck KGaA) were prepared at 5.0 M in sterile distilled water immediately before each experiment.

The solutions of hydrogen peroxide (H_2_O_2_) (Merck KGaA) were prepared by adjusting the concentration of the stock solution at 30% to the final concentrations of 2.0, 5.0, and 9.0% used in the experiments.

#### 4.4.3. Irradiation Conditions

The aPDT assays were carried out under artificial white light conditions. LED projectors (20 W of power, ~230 V of voltage, and with a frequency of ~50 Hz) (EL^®^MARK Holding SE) were used. Light irradiance was adjusted to 50 mW cm^−2^ and measured with a laser power and energy meter FieldMaxII-TOP combined with a high-sensitivity thermopile sensor PS19Q (Coherent, Santa Clara, CA, USA).

#### 4.4.4. aPDT Assays in PBS

The efficiency of the Form at different concentrations (5.0 and 10 μM) was evaluated through quantification of the number of bacteriophages in PBS. Phage phT4A at a concentration of 10^8^ PFU mL^−1^ were tenfold diluted in PBS and distributed in sterilized glass beakers.

The appropriate volume of Form was added to the samples to achieve a final concentration of 5.0 or 10 μM. In these experiments, two controls were simultaneously performed: light control (LC) and dark control (DC). The LC included the bacteriophage suspension subjected to the same light conditions as the samples; the DC included the bacteriophage suspension plus Form at the same concentration as the samples but protected from light (wrapped in aluminum foil) during the assays. Samples and controls were remained in the dark under stirring for 10 min at room temperature to promote the PS binding to the viral particles, before each assay. Then, samples and light controls were exposed to light at 50 mW cm^−2^, for a total period of 270 min of irradiation. Aliquots of samples and controls were collected at intermediate times of light exposure, tenfold diluted in PBS and drop plated (5.0 µL), in duplicate, in Petri dishes previously prepared with TSA and a layer of TSA-soft with the bacteriophage host *E. coli*. The Petri dishes were incubated at 37 °C for 12 h and the number of lysis plaques was counted. The results were expressed as PFU mL^−1^. Three independent assays, with two replicates, for each condition, were performed.

#### 4.4.5. aPDT Assays Performed in Filtered Wastewater

To evaluate the influence of the organic matter present in WW in the aPDT efficiency, assays with wastewater filtered by three different pore size membranes (0.22, 0.30, and 0.45 µm) were carried out.

The aPDT assays in filtered WW by 0.45 μm pore-size membrane were done with different concentrations of Form (1.0, 2.0, 3.0, 4.0, 5.0, and 10 μM) and were tested in the absence of KI (Form) and its presence at 100 mM (Form + KI). The aPDT assays in filtered WW using membranes of pore size 0.22 and 0.30 μm were performed at just one Form concentration (10 µM) in the absence of KI.

The bacteriophage suspension (at a concentration of 10^8^ PFU mL^−1^) was tenfold diluted in 0.45 μm filtered WW and distributed in a sterile 96-well microplate. The appropriate volumes of Form or Form plus KI were added to the samples to achieve a final concentration of Form at 1.0, 2.0, 3.0, 4.0, 5.0, and 10 μM and KI at 100 mM. Once again, dark and light controls were carried out during the aPDT assays (at the same conditions as described above). A pre-irradiation period of 10 min in the dark, at room temperature, and under stirring was carried out and then samples and light controls were exposed to artificial light with an irradiance of 50 mW cm^−2^. Aliquots of the samples and controls were collected at predefined times of light exposure. The bacteriophage suspensions were serially diluted in PBS and plated with their hosts, as previously described, by the drop plated method. The Petri dishes were incubated at 37 °C for 12 h and the number of lysis plaques was counted, and the obtained results were expressed as PFU mL^−1^. At least three independent assays, with two replicates, for each condition, were performed.

Similar conditions were used in the assays performed using the WW filtered by the membranes of pore size 0.22 and 0.30 μm (Form at 10 μM in the absence of any adjuvant) to investigate the impact of the organic matter content of the aqueous matrix on the aPDT outcomes.

#### 4.4.6. aPDT Assays Performed in Non-Filtered Wastewater

To assess if the efficiency of aPDT to inactivate bacteriophages was maintained in raw WW, assays with Form at 5.0 and 10 μM were performed. In this medium, the KI and H_2_O_2_ potentiators’ effect on aPDT efficiency was also evaluated. Thus, assays were performed with Form (at concentrations of 5.0 and 10 μM) with KI at 100 (for Form at 5.0 and 10 μM) and 200 mM (for Form at 10 μM) (Form + KI) for a period of irradiation of 15 min (sublethal doses), followed by dark incubation to a total of 24 h post-irradiation. Similar assays were also performed in the presence of H_2_O_2_ at 2.0, 5.0, and 9.0% (Form + H_2_O_2_). These assays were prepared according to the procedure described above. Aliquots of the samples and controls were collected at defined times of light exposure and the bacteriophage concentration was quantified as described above. Three independent assays, with two replicates, for each condition, were performed.

#### 4.4.7. Evaluation of Longer-Lived Reactive Species Generated during aPDT

Assays in PBS, with 0.45 µm filtered WW, were also performed to test if longer-lived reactive species generated when aPDT was done in the presence of KI [e.g., free iodine/triiodide (I_2_/I_3_^−^), iodine radicals (I_2_^•−^), and hydrogen peroxide (H_2_O_2_)] were able to continue reducing the content of viruses in the sample after the light treatment. These aPDT assays were performed in PBS with Form at 5.0 and 10 µM and the addition of KI at 100 mM. In these assays, for the two tested Form concentrations after 15 min (sublethal dose), aliquots were collected immediately after the irradiation periods; then, the samples were incubated in the dark at room temperature and more aliquots were collected after defined periods of dark incubation (60, 180, and 360 min, and 24 h). Aliquots of the samples and controls were collected at defined times of light exposure and the bacteriophage concentration was quantified as described above. Assays with 0.45 µm filtered WW for the evaluation of longer-lived reactive species, generated during aPDT treatment in the presence of KI, were also performed using Form at 5.0 µM and the addition of KI at 100 mM. In these assays, two sublethal irradiation periods (15 and 30 min of irradiation) were selected. For the samples irradiated for 15 min, aliquots were collected immediately after the 15 min of irradiation (0 min post-irradiation) and after 15, 30, 45, 60, and 90 min of post-irradiation in dark incubation. For the ones irradiated during 30 min, fewer time-spaced aliquots were taken—immediately after the irradiation period (0 min post-irradiation) and after 5, 10, 15, 30, 45, 60, and 90 min of dark incubation. Aliquots of the samples and controls were collected at defined times of light exposure and the bacteriophage concentration was quantified as described above. At least three independent assays, with two replicates, for each condition, were performed.

### 4.5. Statistical Analysis

The statistical analysis was performed on the data resultant of three independent assays done in duplicate for each condition tested. The statistical analysis was done with GraphPad Prism. Normal distributions were checked by the Kolmogorov–Smirnov test. ANOVA and Tukey’s multiple comparisons test was applied to assess the significance of the differences between the bacteriophage concentration along with the aPDT treatments. A *p* value < 0.05 was considered to be statistically significant.

## 5. Conclusions

The emergence of highly pathogenic microorganisms has serious health risks, as shown last year (more significantly for SARS-CoV-2), where incorrect disinfection of WW led to the detection of these pathogens in the receiving water bodies (where WW are released), and, eventually, became a vehicle for their transmission to humans [103,104]. As previous studies demonstrated, and the present study corroborated, aPDT is a technique that has been shown to be highly efficient in inactivating microorganisms, including viruses, in highly heterogeneous media (e.g., secondary treated wastewater), even when using low PS concentrations (5.0–10 µM). Overall, photodynamic therapy with Form even if used alone was shown to be efficient in viruses’ inactivation in secondarily treated WW. The addition of H_2_O_2_ during aPDT with Form potentiates the virus inactivation performance, allowing a significant reduction in PS concentration and treatment time. The results obtained in this study support that aPDT can overcome the disadvantages that other WW tertiary disinfection techniques have, both in their application (costs, manpower, infrastructure, etc.) and for the organisms in the receiving waters, thus proving to be a promising and safe option for efficient WW tertiary treatment before it is released in nature. This alternative approach would help surpass the disadvantages of chlorine used as one of the most-used methods of water disinfection, namely the formation of by-products with potential health risks as a consequence of its reaction with organic compounds. Additionally, future studies must be conducted in order to improve knowledge on the possible effects of organic matter on aPDT efficiency. Moreover, other interesting steps to be done is to test the PS immobilized in some supports (enabling the recovery of the PS after use) followed by the evaluation of the performance of the presented protocol in a pilot scale test, under solar light.

## Figures and Tables

**Table 1:** Schematic representation of possible positive effects that the presence of organic matter (OM) in the aPDT matrix (WW) may have on the antimicrobial inactivation efficiency.

PS+ hν→P1S*→ISCP3S*	PS absorption of a photon of energy from light
P3S*+Subs →e−transfer oxygen radicals (O2•−, H2O2, HO•)	Type I mechanism, electron transfer pathway
P3S*+O32→energy transfer PS+O12	Type II mechanism, energy transfer pathway
P3S*+OM→ PS+ reactive oxidation products	Interactions of oxygen radicals and/or ^1^O_2_ with OM present in the WW matrices
OM+O12→ reactive oxidation products	

## Data Availability

Not applicable.

## Supplementary Materials

The following are available online at https://www.mdpi.com/article/10.3390/antibiotics10070767/s1, Figure S1: Effect of residual iodine species in dark incubation after aPDT assay in T4-like bacteriophage in buffer PBS with Form 5.0 (A) and 10 µM (B) and KI at 100 mM, during 15 min of irradiation with white light (50 mW cm^−2^); Figure S2: Effect of residual iodine species in dark incubation after aPDT assay in T4-like bacteriophage in filtered WW (0.45 µm filtration) with Form at 5.0 µM and KI at 100 mM, during 15 (A) and 30 min (B) of irradiation with white light (50 mW cm^−2^).
