# Supplementary material for: Antimicrobial Photodynamic Approach in the Inactivation of Viruses in Wastewater: Influence of Alternative Adjuvants
Source: Antibiotics (Basel). 2021 Jun 24;10(7):767. doi: 10.3390/antibiotics10070767 (PMC8300698; doi:10.3390/antibiotics10070767)
Supplement: Supplementary file 1 [file antibiotics-10-00767-s001.zip › antibiotics-1257420-supplementary.pdf]

antibiotics

MDPI

Supplementary Material for

Article

Antimicrobial Photodynamic Approach in the Inactivation of Viruses in Wastewater: Influence of Alternative Adjuvants

Maria Bartolomeu [1], Cristiana Oliveira [1], Carla Pereira [1], M. Graça P. M. S. Neves [2], M. Amparo F. Faustino [2,*] and Adelaide Almeida [1,*]

[1] Department of Biology and CESAM, University of Aveiro, 3810-193 Aveiro, Portugal; maria.bartolomeu@ua.pt (M.B.); cristianapoliveira@ua.pt (C.O.); csgp@ua.pt (C.P.)

[2] Department of Chemistry and LAQV-REQUIMTE, University of Aveiro, 3810-193 Aveiro, Portugal; gneves@ua.pt

* Correspondence: faustino@ua.pt (M.A.F.F.); aalmeida@ua.pt (A.A.); Tel.: +351-234-401-406 (M.A.F.F.); +351-234-370-784 (A.A.)

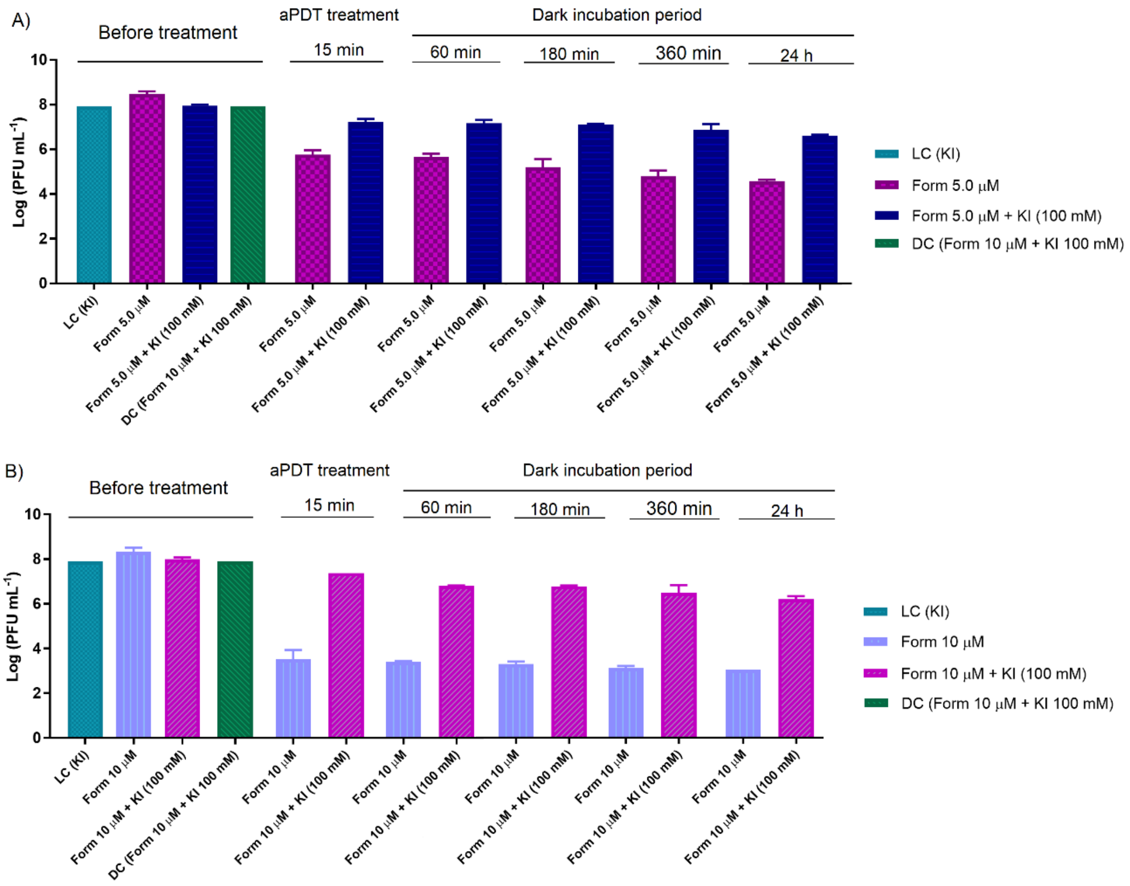

Figure S1. Effect of residual iodine species in dark incubation after aPDT assay in T4-like bacteriophage in buffer PBS with **Form** 5.0 (**A**) and 10 μM (**B**) and KI at 100 mM, during 15 min of irradiation with white light (50 mW cm^{-2}). The values are expressed as the mean of three independent experiments; error bars represent the standard deviation (SD) between the experiments. In some cases, SD bars are covered behind the bars.

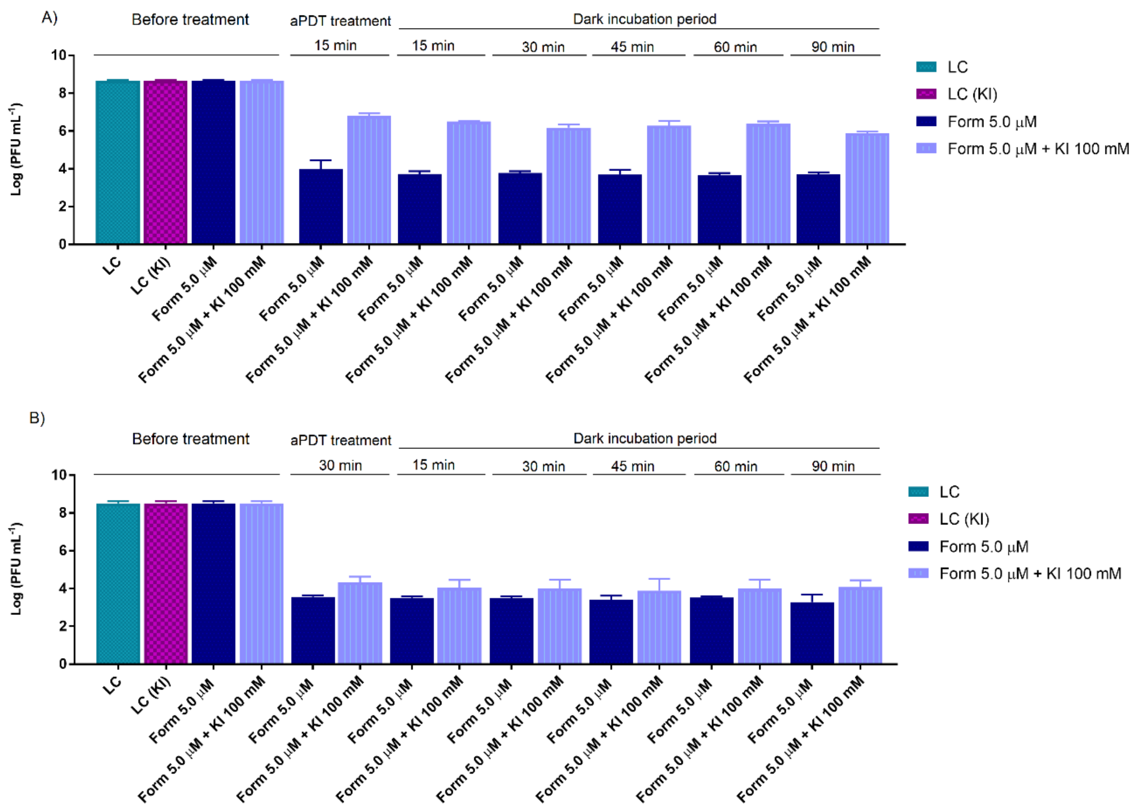

Figure S2. Effect of residual iodine species in dark incubation after aPDT assay in T4-like bacteriophage in filtered WW (0.45 µm filtration) with **Form** at 5.0 µM and KI at 100 mM, during 15 (**A**) and 30 min (**B**) of irradiation with white light (50 mW cm^{-2}). The values are expressed as the mean of three independent experiments; error bars represent the standard deviation (SD) between the experiments. In some cases, SD bars are covered behind the bars.
